# Compressive Load Capacity of Concrete Structures Made of Hollow Blocks with Voids Filled with Concrete of Various Features

**DOI:** 10.3390/ma17246262

**Published:** 2024-12-21

**Authors:** Vadim Griniov, Kseniya Yurkova, Rafał Prusak

**Affiliations:** 1 Faculty of Civil and Environmental Engineering, Warsaw University of Life Sciences, Nowoursynowska St. 166, 02-787 Warsaw, Poland; vgvfima@gmail.com; 2 Faculty of Civil Engineering, Czestochowa University of Technology, Dabrowskiego 69, 42-201 Czestochowa, Poland; 3 Faculty of Production Engineering and Materials Technology, Czestochowa University of Technology, Dabrowskiego 69, 42-201 Czestochowa, Poland; rafal.prusak@pcz.pl

**Keywords:** masonry prisms, hollow concrete blocks, concrete infill, compressive load capacity, finite element method

## Abstract

A masonry made of hollow concrete blocks in modern constructions differs from the traditional one in that the empty space (up to 70%) makes it possible to create complex high-strength load-bearing structures by filling the voids with monolithic or reinforced concrete. The aim of this study was to examine specimens of concrete structures made of hollow blocks with voids filled with concretes with various features. The research methodology is based on the results of numerical and experimental tests. Laboratory studies were conducted to determine the influence of the concrete filling on strength, deformability and the nature of destruction of the experimental specimens. The numerical analysis was performed on the basis of FEM with the use of the ANSYS 2021 R1 software. The method for determining the load capacity of multi-component structures using strain diagrams of constituent materials has been improved. There is strong agreement between the numerical and experimental results for all masonry prisms. Additionally, a good correlation was observed between the experimental results and the analytical calculations performed using the proposed methodology.

## 1. Introduction

With a very high level of construction intensity taking place in EU countries over the past 5–10 years, the requirements for residential and industrial buildings are increasing. The cost of erecting walls of a building amounts to 30–50% of the cost of the entire building, with the main building material for the construction of walls being bricks and blocks. The blocks can be made of various materials (concrete, ceramics) and can be hollow or solid. Large geometric dimensions and various types of voids allow for an increase in the pace of construction and the application of hollow concrete blocks (HCBs) as a universal building element in the construction of load-bearing walls, columns and lintels.

**Figure 1:**
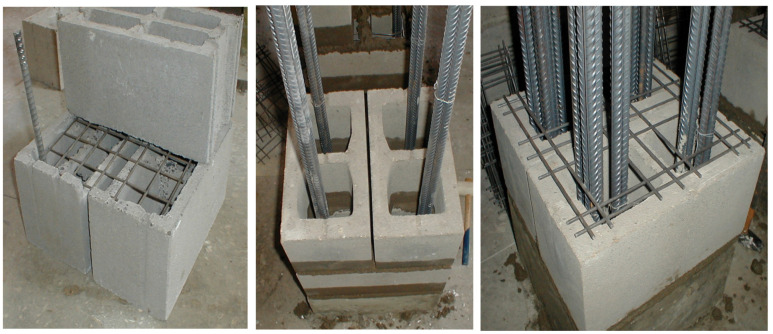
Vertical and horizontal reinforcement of HCB walls.

The presence of vertical voids in the walls of structures made of hollow blocks allows them to be filled with monolithic or reinforced concrete. This technology makes it possible to create structural systems of buildings with increased strength and rigidity (Figure 1).

The complexity of calculating these structures lies in the interaction between three materials, or four if a reinforcement is present: the HCB, a mortar between blocks, the concrete infilling voids and a steel reinforcement. It is also difficult to determine which regulatory document refers to the specified structure concerning masonry or concrete structures.

Experimental and theoretical studies of fragments of walls and prisms made of HCBs have been carried out by scientists in different countries.

Empirical formulas for calculating the strength of masonry made of HCBs were proposed in [1]. The authors conducted an experiment with different block strengths. In [2], tests were conducted on masonry prisms consisting of two rows of blocks, and the effect of time on strength and Young’s modulus was studied. The analysis in [3] describes experiments on testing masonry walls, considering the influence of various parameters. In [4], an experiment testing HCBs with different strengths is described, along with the mechanism through which the properties of blocks and mortars influence the compressive strength of masonry.

Practical recommendations for the calculation of masonry are presented in the works in [5,6,7,8].

The results of the effective use of the method of filling voids of concrete blocks with concrete and metal reinforcement are presented in [9,10,11]. In the papers [12,13], the effect of mortar quality on the load capacity of HCB prisms was studied and the nature of the failure was given. In [14], the modulus of elasticity was determined during the test on HCB prisms, and computer simulations were carried out. In work [15], a numerical study of a 3D model of reinforced concrete prisms under tensile testing was conducted. The works [16,17] confirm the cooperation between the concrete infill and hollow blocks, providing a description of the failure of specimens as well. In [18,19], a model for analyzing structures made of blocks with different mortars and shapes of voids was proposed, and a 3D model of HCB masonry prisms was developed.

Regulatory requirements for the calculations of the structures being studied are presented in the Eurocodes [20,21,22,23]. In Eurocode Section 3.6.1.2 (4) [20], for calculations of masonry with vertical cavities filled with concrete, it is recommended to apply the lower strength from among the following: the strength of HCB masonry or the strength of a concrete infill. In Eurocode Section 6.6.1 (4) [20], for reinforced masonry elements with a concrete infill, it is recommended that when the compression zone consists of masonry and concrete, the compressive strength should be calculated based on the stresses of the weakest material. The specified recommendations of paragraphs 3.6.1.2 (4) and 6.6.1 (4) [20] limit the use of concrete for filling voids if its strength exceeds the strength of the HCB masonry.

For modeling and computer calculations of masonry structures, a stress–strain relationship is required. The authors believe that the absence of this relationship for HCB masonry prisms in the existing regulatory documents represents a significant gap.

The objectives of the research in this work are as follows:

- To study the stress distribution and type of failure of masonry prisms with and without concrete infill, under axial compression;
- To determine the effects of the mechanical properties of the components making up the prism (HCB, mortar and concrete infill) on its load capacity and deformability;
- To select the optimal stress–strain relationship for the masonry prisms;
- To apply the finite element method for a detailed description of the stress distribution in the axially compressed prisms.

## 2. Experimental Program, Materials and Specimens

**Figure 2:**
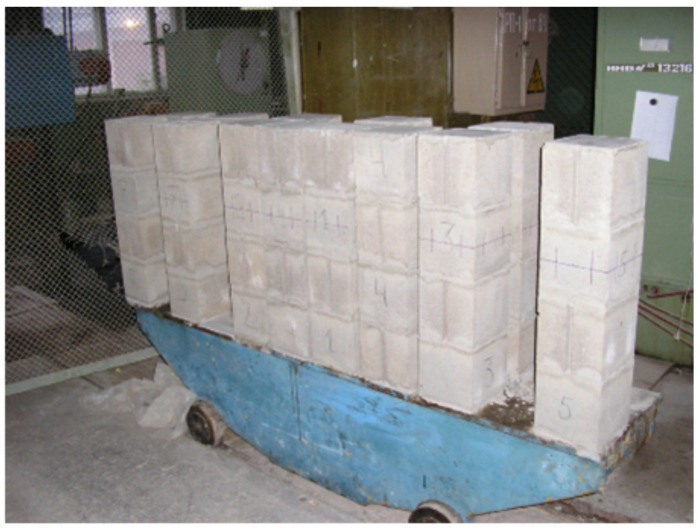
Masonry prisms.

**Figure 3:**
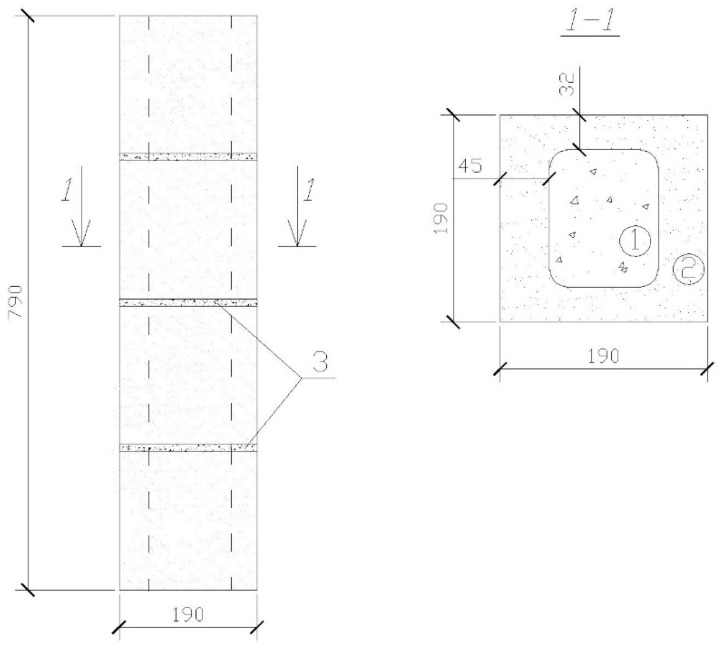
Structure of the prisms: 1—concrete infill; 2—HCB; 3—mortar.

To achieve the above objectives, tests of HCB masonry prisms with and without a concrete infill were carried out. The experimental masonry prisms were made of four rows of HCBs (Figure 2 and Figure 3).

After obtaining the strength of the mortar, the voids of the prisms were filled with a concrete mixture. Various concrete compositions were used to fill the HCB masonry prisms. Three specimens were produced in each of the seven series of specimens (P1–P7); the specimens of the P7 series were not filled with concrete (Table 1).

**Figure 4:**
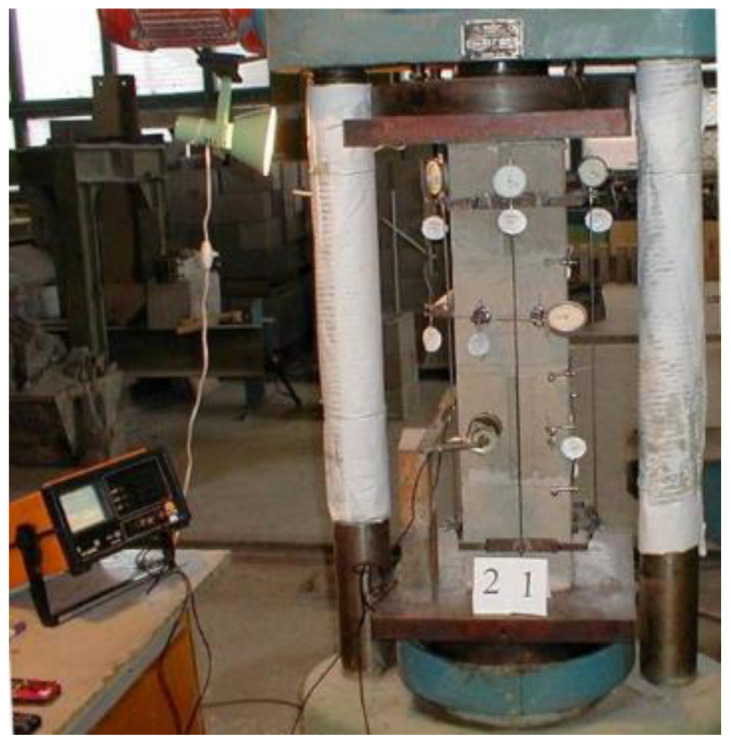
Testing of specimen P2.

**Figure 5:**
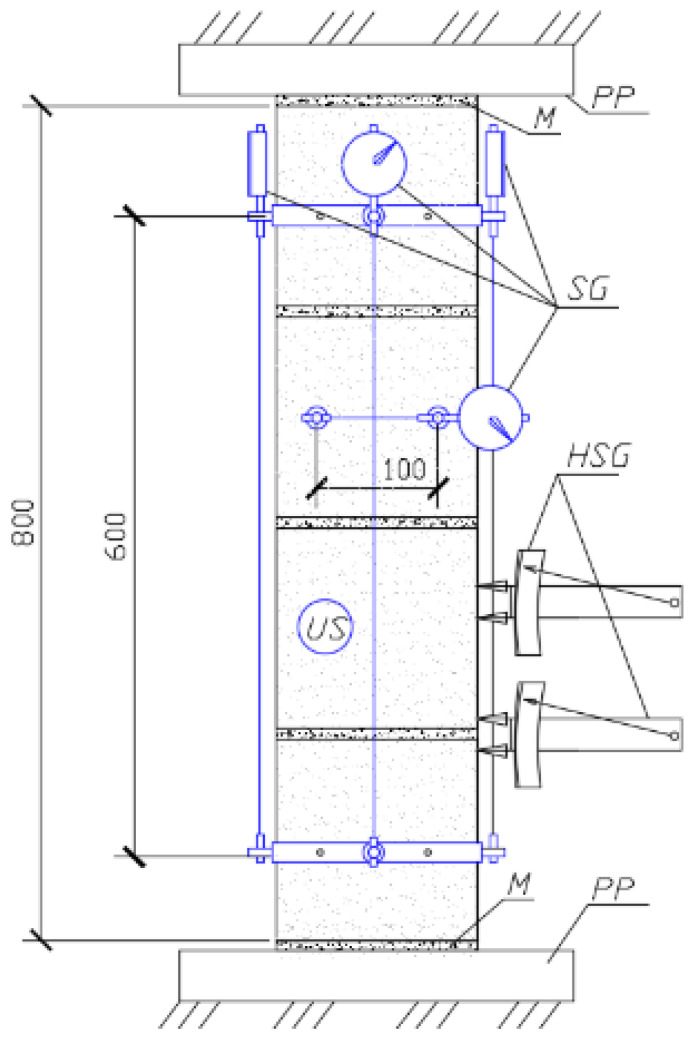
Layout of measuring instruments on specimens P1–P7: SG—dial gauge; HSG—axial extensometer; US—sounding converter; M—leveling mortar layer; PP—press base plates.

Testing of the specimens after gaining strength in natural laboratory conditions was carried out after at least 28 days in a hydraulic press R-125, according to a stepwise short-term loading mode recommended by DIN 18554 p. 1 (3.2) [24]. At each load level, the following quantities were measured: longitudinal strains using a dial gauge (measuring base: 600 mm, accuracy 0.01 mm); transverse strains using a dial gauge (measuring base: 100 mm, accuracy 0.001 mm); longitudinal strains of the mortar zone using an axial extensometer (measuring base: 20 mm, accuracy 0.001 mm). By changing the speed of ultrasound passage through the thickness of the prism, the continuity and quality of contact between the inner surface of the block and the concrete infill were determined (Figure 4 and Figure 5).

## 3. Results of the Conducted Research

### 3.1. Nature of the Failure and Load Capacity of the Specimens

**Figure 6:**
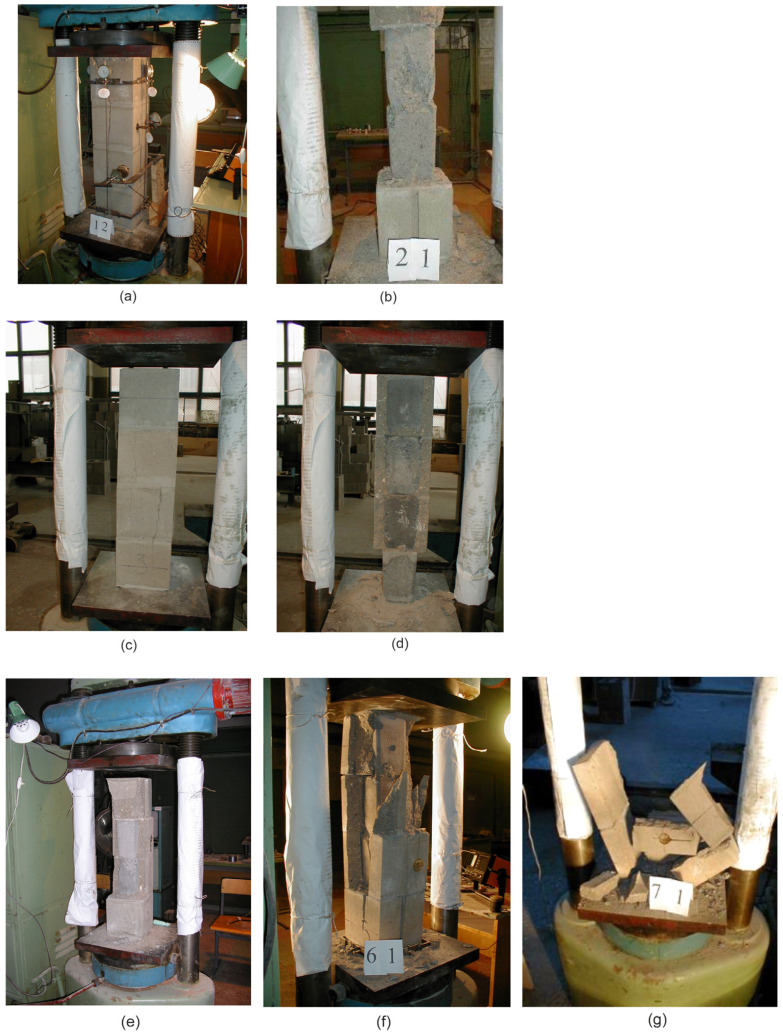
Failure of prisms: (**a**) specimen P1; (**b**) specimen P2; (**c**) specimen P3; (**d**) specimen P4; (**e**) specimen P5; (**f**) specimen P6; (**g**) specimen P7.

**Figure 7:**
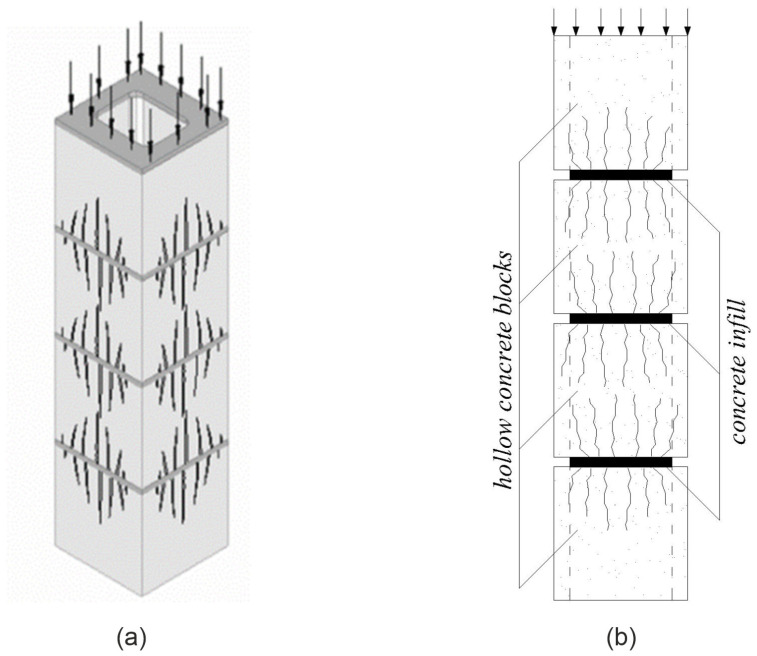
Crack patterns: (**a**) masonry prisms without filling; (**b**) masonry prisms with filling.

Figure 6 and Figure 7 show the nature of the failure of the HCB masonry prisms without a concrete infill (a) and prisms filled with concrete (b) under the action of axial force.

HCB masonry prisms without a concrete filling collapsed in a brittle way. The hollow concrete blocks have greater rigidity than the connecting mortar, and the transverse expansion of the mortar increases the tensile stresses in the walls of the hollow block (double Poisson effect). Tensile stresses lead to the appearance of vertical cracks starting from the place of contact between the block and the mortar. As the vertical load increases, cracks grow along the height, and the block divides into separate fragments, which is followed by a loss of stability and brittle destruction.

HCB masonry prisms filled with concrete did not fail in a brittle manner, the cracks—as in the prisms without filling—began to grow in the vicinity of the contact between the block and the mortar. The smooth failure of the HCB masonry prism with a concrete infill is caused by its restraining effect. The concrete filling voids have greater rigidity compared to that of the masonry prism, and the lower rigidity of the masonry prism is caused by significant strains on the mortar in their contact zone.

The axial compressive load capacity of the masonry prisms is shown in Table 2.

### 3.2. Strains of Experimental Concrete Specimens

The values of strains and the nature of their development are the key data for the analysis of structures at all stages of testing. The adopted measurement technique made it possible to obtain values of longitudinal and transverse strains at all these stages. Longitudinal strains of specimens refer to average strains measured along the height of the specimen in the areas of the predicted damage, taking horizontal joints into account.

Table 3 shows the maximum longitudinal strains measured on the surfaces of the concrete block ($\varepsilon_{1}$) and the contact zone of the block with the mortar ($\varepsilon_{3}$) along the height of the prism ($\varepsilon_{2}$) and the transverse strains of the concrete block ($\varepsilon_{ct}$), which correspond to the maximum load.

**Figure 8:**
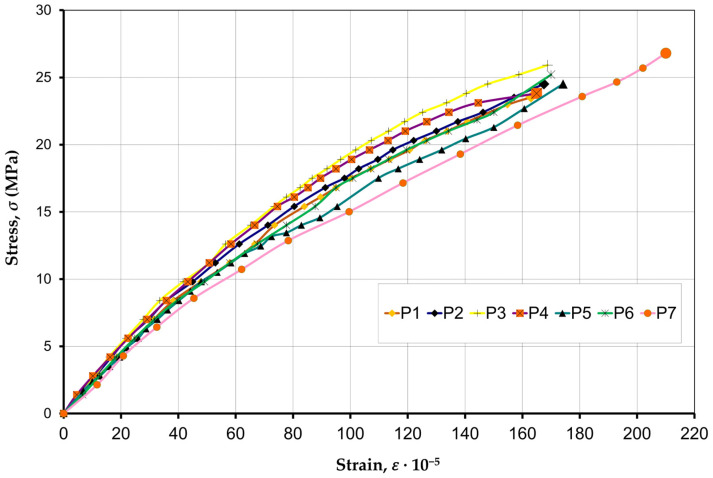
Compression stress–strain curves of specimens P1–P7.

The nature of the development of longitudinal strains ($\varepsilon_{2}$) (Figure 8) showed that they have a slight dispersion, $170\cdot 10^{-5}–190\cdot 10^{-5}$. The study of the deformations of specimens P1–P6 did not reveal significant differences for prisms with different compositions of concrete infill. Stress–strain curves for the specimens filled with concrete have a higher curvature compared to the specimens without an infill.

**Table 3:** Strains on local sections of the prism.

Specimen	$\varepsilon_{1}\cdot 10^{-5}$, (Zone B, Figure 9)	$\varepsilon_{3}\cdot 10^{-5}$, (Zone A, Figure 9)	$\varepsilon_{2}\cdot 10^{-5}$, (Zone C, Figure 9)	$\varepsilon_{ctu}\cdot 10^{-5}$, Maximum Transverse Strain
P1	120	700	170	20
P2	110	740	180	22
P3	110	870	190	20
P4	120	550	185	20
P5	120	800	170	19
P6	100	650	170	23
P7	110	1050	210	18

**Figure 9:**
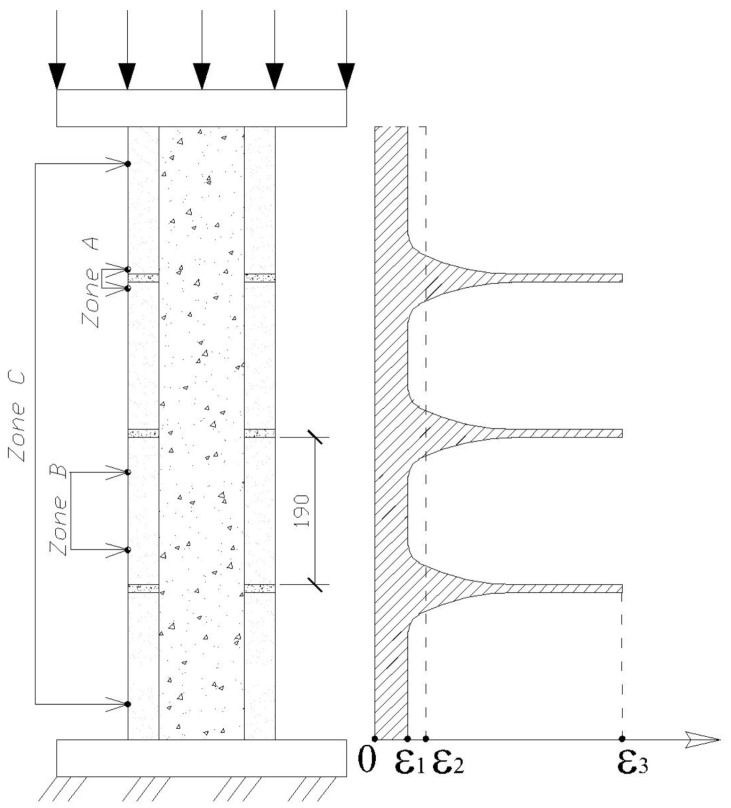
A diagram of longitudinal strains in different zones: $\varepsilon_{1}$—HGB (zone B); $\varepsilon_{2}$—masonry prism (zone C); $\varepsilon_{3}$—mortar (zone A).

**Figure 10:**
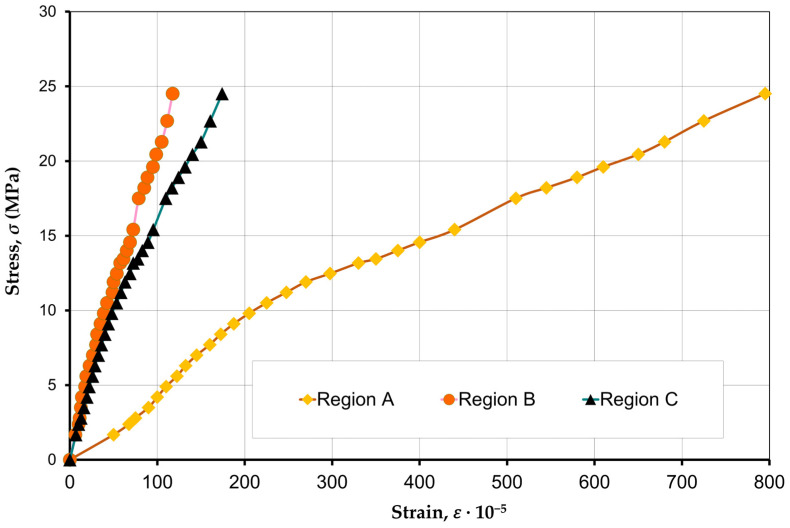
Compression stress–strain curves in local sections of the masonry prism.

Measurements of strains along the height of the specimens showed that the mortar zone has the greatest deformability. The maximum strains are $\varepsilon_{3}=1050\cdot 10^{-5}$ in specimen P7 and the minimum strains are $\varepsilon_{3}=650\cdot 10^{-5}$ in the prisms filled with concrete. The heterogeneous distribution of the longitudinal strains in sections along the height of the prism is shown in Figure 9. The nature of the development of longitudinal strains of different sections during the loading of the masonry prism is shown in Figure 10.

To determine the strains of different sections of the masonry prisms, Formula (1) is proposed

(1) $$\;\varepsilon_{2}=\frac{\varepsilon_{1}\cdot \left(l-n\cdot t\right)+\varepsilon_{3}\cdot \left(n\cdot t\right)}{l},$$

where $\varepsilon_{1},\varepsilon_{2},\varepsilon_{3}$ are strains of concrete blocks, masonry prisms and mortar sections, respectively; *l* is the specimen length; n is the number of mortar joints; and *t* is the mortar thickness.

Based on the experimental results and the literature data [25,26], three Formulas (2)–(4) have been proposed to describe the stress–strain relationship for the materials used:

- a logarithmic Equation (2) [25]:

(2) $$\;\;\sigma \left(\varepsilon_{m,c}\right)=\mu \cdot f_{m,c}\cdot \left[1-e^{\left(-0.9\cdot \varepsilon_{m,c}\cdot \alpha \right)}\right];$$

- parabolic equations by Hognestad (3) [26] and Tomaczewicz (4):

(3) $$\sigma \left(\varepsilon_{m,c}\right)=f_{m,c}\cdot \left[2\cdot \left(\frac{\varepsilon_{m,c}}{\varepsilon_{m,c.max}}\right)-{\left(\frac{\varepsilon_{m,c}}{\varepsilon_{m,c.max}}\right)}^{2}\right],$$

(4) $$\sigma \left(\varepsilon_{m,c}\right)=f_{m,c}\cdot \left[\frac{\beta \cdot \frac{\varepsilon_{m,c}}{\varepsilon_{m,c.max}}}{\beta -1+{\left(\frac{\varepsilon_{m,c}}{\varepsilon_{m,c.max}}\right)}^{\beta }}\right]$$

where α=1500, μ=1 — coefficients; $\beta =f_{m,c}/20$—material parameter; $f_{m,c}$—maximum strength of masonry prism or the concrete infill (MPa); $\varepsilon_{m,c.max}$ and $\varepsilon_{m,c}\;$— strains corresponding to the maximum and intermediate strength of materials.

**Figure 11:**
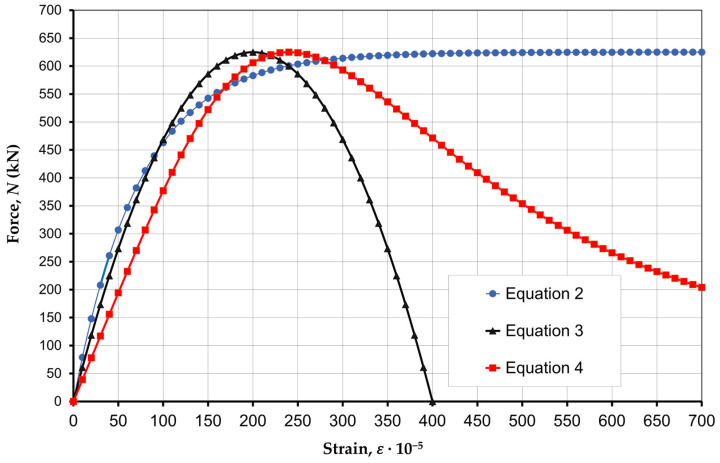
Compression force–strain curves of the masonry for prism P7.

Figure 11 shows the force–strain curves based on Equations (2)–(4) for the HCB masonry prism without a concrete infill—P7.

A comparison of experimental data with Equations (2)–(4) showed that the nature of strains during the axial compression of the masonry prism corresponds to the Hognestad Equation (3), and the nature of concrete strains corresponds to the Tomaczewicz Equation (4). Equation (2) has a logarithmic character but its use in calculations leads to large errors, so it will not be used in load capacity calculations.

## 4. Load Capacity of HCB Masonry Prisms

This research paper considers two methods for calculating the load capacity of the HCB masonry prisms filled with concrete. Both methods use individual stress–strain curves for the HCB masonry prism and the concrete infill:

- The first method, according to the current EU standards 3.6.1.2(4) [20], where the calculation uses the minimum strength of one of the two components (HCB masonry prism or concrete infill), is used to calculate the entire section.
- The second method involves summing the load capacity of concrete with that of a masonry prism and using individual strength characteristics for each component.

During axial compression, the masonry prism deforms together with the concrete infill:

(5) $$\varepsilon_{m}=\varepsilon_{c}$$

where $\varepsilon_{m}$ and $\varepsilon_{c}$ are strains of the HCB masonry prisms and concrete infill, respectively.

Condition (5) allows calculations when the stress–strain curve for the HCB masonry prism filled with concrete is given.

The equilibrium condition for calculating the load capacity of a masonry prism is taken as Formula (6):

(6) $$\;\;N=\sigma_{c}\left(\varepsilon \right)\cdot A_{c}+\sigma_{m}\left(\varepsilon \right)\cdot A_{m}$$

where N—the load capacity of the HCB masonry prism with the concrete filling; $A_{c}$—the cross-section area of the concrete infill; $A_{m}$—the area of the HCB masonry prisms; $\sigma_{c}\left(\varepsilon \right)$—a stress–strain function for the concrete infill, determined by the Tomaczewicz Equation (4); and $\sigma_{m}\left(\varepsilon \right)$—a stress–strain function for the HCB masonry prisms, determined by the Hognestad Equation (3).

The calculation is based on Equations (3) and (4), establishing a relationship between stresses and strains for the concrete infill and masonry prisms. The calculation of the masonry prisms P1 is performed using two methods.

According to the first method, the load capacity of the prism N1 is equal to 496 kN, wherein stresses in the infill concrete $f_{c}$ = 14 MPa were applied (Table 1). Also, according to the EU recommendations 6 p. 3.6.1.2 (4) [20], the stresses in the masonry prisms were compared to the concrete stresses $f_{m}$ = $f_{c}$ = 14 MPa, since $f_{m}>f_{c}$.

According to the second method, the load capacity of the prism N2 is equal to 794 kN, wherein stresses in the infill concrete $f_{c}$ = 14 MPa were applied (Table 1), and the stresses of the masonry prism $f_{m}$ = 27 MPa were compared (Table 2).

**Figure 12:**
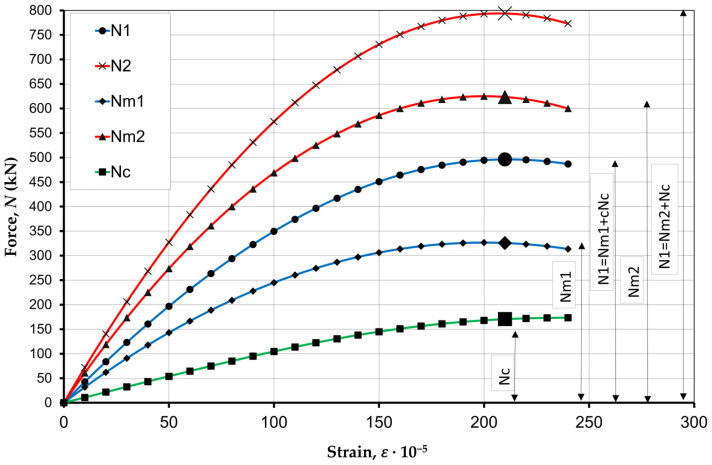
The load capacity of the masonry prism P1 according to two calculation methods.

To graphically compare the calculation results obtained with the use of these two methods, curves of force distribution between the compressed components of the cross-section (HCB masonry prism and concrete infill) of specimen P1 were created (Figure 12).

Here, N1 is the prism load capacity calculated according to the method 1; N2 is the prism load capacity calculated according to the method 2; Nm1 is the load capacity of the masonry prism without a concrete infill according to the method 1; Nm2 is the load capacity of the masonry prisms without a concrete infill according to the method 2; and Nc is the load capacity of the concrete infill. The curves show that the strength of the concrete-filled masonry prisms calculated by method 1 is underestimated compared to method 2.

As shown by the comparison of the experimental and theoretical values of the load capacity of the samples (Table 4), the second calculation method shows the best convergence with the experimental data; the error is between 2% and 6%. In the first method, the error rate was up to 69% compared to the experimental data.

## 5. Numerical Experiments

The modeling of test specimens is an important part of experiments. The ANSYS 2021 R1 software, which implements the finite element method, was used for digital simulations of the tests. In the calculations, the material was modeled using eight-node SOLID185 elements and the elastic-plastic Menetrey–Willam (MW) material model with hardening/softening in compression and softening in tension (HSD2 model), implemented in the ANSYS program [27,28]. The parameters for modeling the material of hollow concrete blocks, the mortar and the concrete infill were determined based on the known properties of these materials used to make specimens (Table 1). The boundary conditions were defined such that the lower base of the prism was fixed along the direction of its longitudinal axis, while in the orthogonal directions the constraints were applied according to the symmetry conditions.

**Figure 13:**
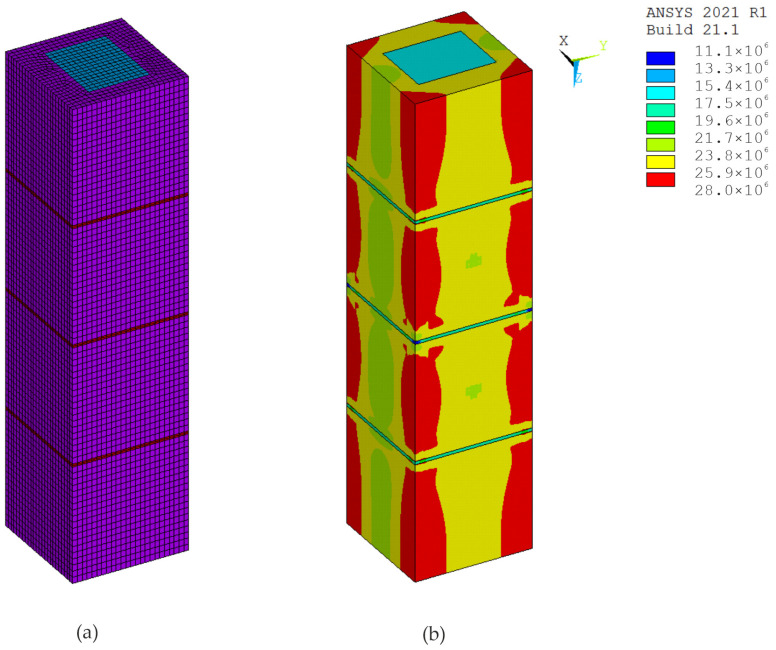
Specimen P1: (**a**) calculation scheme; (**b**) map of equivalent stress resulting from axial force (Pa).

**Figure 14:**
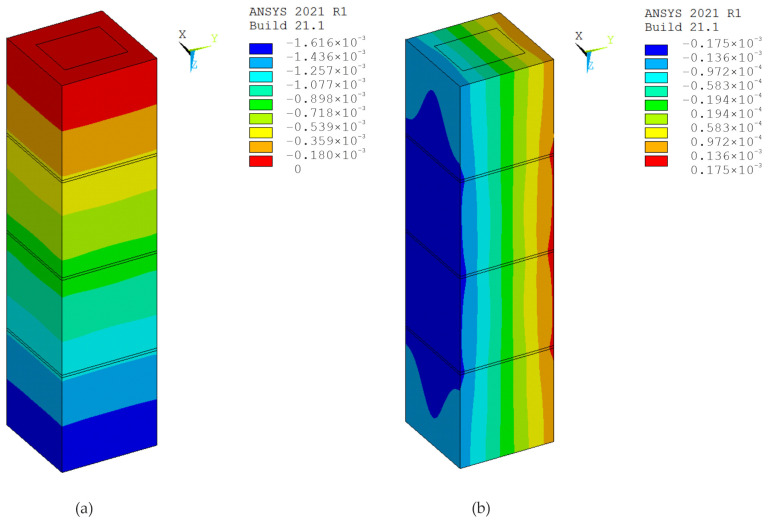
Displacement maps with axial force, specimen P1: (**a**) longitudinal (m); (**b**) transverse (m).

**Figure 15:**
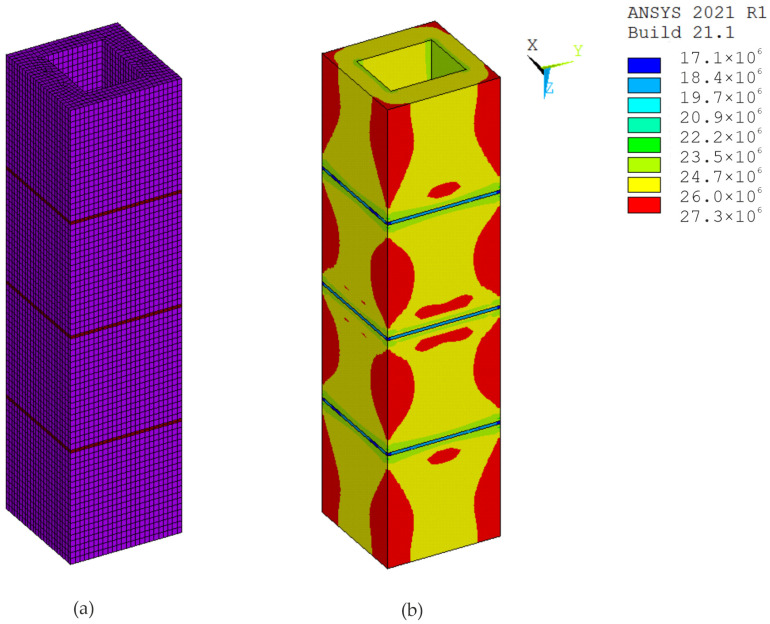
Specimen P7: (**a**) calculation scheme; (**b**) map of equivalent stress resulting from axial force (Pa).

**Figure 16:**
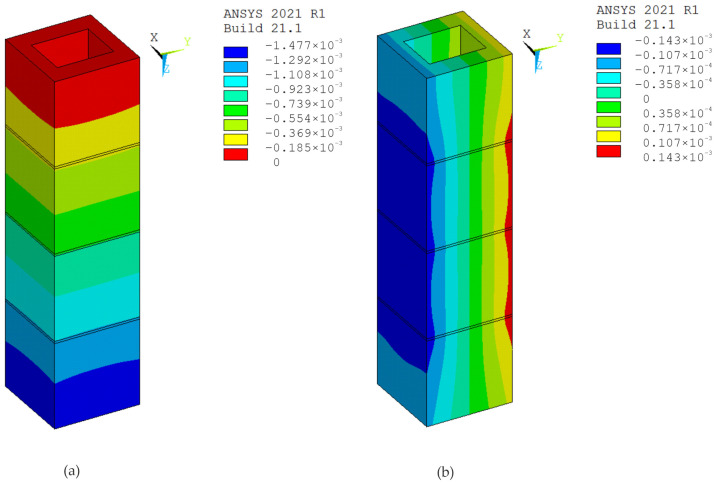
Displacement maps with axial force, specimen P7: (**a**) longitudinal (m); (**b**) transverse (m).

Simulations and computer testing of the HCB masonry prisms with an infill are presented in Figure 13 and Figure 14, and the results of the tests on HCB masonry prisms without a concrete infill are presented in Figure 15 and Figure 16. If an axial load of 800 kN is exerted on specimen P1, the displacement along the Z-axis is 1.62 mm, and the displacements along the X- and Y-axes are 0.035 mm. Calculation schemes with displacement maps are shown in Figure 14. If an axial load of 600 kN is exerted on specimen P7, the displacement along the Z-axis is 1.48 mm, and the displacement along the X- and Y-axes is 0.028 mm. Calculation schemes with displacement maps are shown in Figure 16.

The analysis of merely longitudinal strains does not fully characterize the behavior of the structure; therefore, it was supplemented by the analysis of transversal and local strains in the mortar zone. As it appeared, the local strains in the contact zone of two HCBs have limit values 7–8 times higher than the longitudinal strains measured at the surface of the concrete blocks. The reason for this phenomenon is the mortar having different deformation properties and a lower Young’s modulus than that of the concrete.

With such strain features, the mortar is compressed at the joint, and the tensile stresses reach their maximum values at the points of contact of the concrete blocks with the mortar. The model created in ANSYS (Figure 15b) clearly shows the tensile stresses in the edge of the block (zone 1) in the X and Y direction and compressive stresses in mortar (zone 2) along the Z-axis.

The transverse expansion of the mortar causes tensile stresses in the walls of the hollow block (double Poisson effect), which leads to the appearance of the first cracks.

The numerical and experimental results of stresses and displacements for specimen P1 loaded by 600 kN and specimen P7 loaded by 800 kN are presented in Table 5.

**Figure 17:**
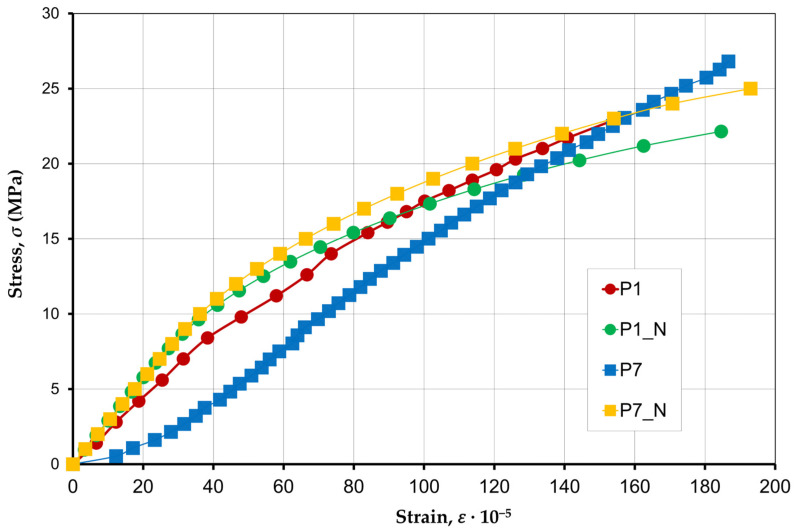
Compression stress–strain curves of specimens P1 and P7 and simulated specimens P1_N and P7_N.

Figure 17 shows a comparison of the stress–strain curves of P1 and P7 obtained during the tests and the curves of P1_N and P7_N related to the numerical results derived from the numerical analyses performed using the specialized finite element software.

The verification of the test results for the specimens in the calculation program showed that the assumed calculation model in ANSYS is adequate and corresponds to the real construction (Figure 17).

## 6. Conclusions

The presented analysis allows for the following conclusions:

- The cooperation of the masonry prisms with the concrete infill has been experimentally confirmed. The relation between transverse and longitudinal strains in the sections of horizontal joints has been established. Due to the development of these strains, longitudinal cracks are being formed and brittle failure occurs in specimens not filled with concrete.
- Filling the voids of masonry prisms allows the maximization of the load capacity characteristics of hollow blocks and limits the tensile forces in the joints of the masonry. The existing regulatory documents (method 1) prevent the use of a concrete filling with strength exceeding that of masonry prisms. The underestimation of the actual strength in calculations using method 1 reaches up to 69%.
- The stress–strain relationship for HCB masonry prisms without a concrete infill was selected. A new simple method for calculating the uniaxial load capacity of the HCB masonry prisms with concrete infill, suitable for practical use, was proposed. The methodology is confirmed by the comparison of the results of our research and the results of tests conducted by other researchers. The calculation error of the proposed method (method 2) is 6%.
- Based on the conducted experimental and numerical analysis with FEM, the authors of the article propose that the load capacity of the related materials should be taken into account when calculating structures made of HCBs with a concrete infill.

## Figures and Tables

**Table 1:** Characteristics of the materials used to make specimens.

Specimen	Strength of Blocks, $f_{b}$ (MPa)	Strength of Mortar, $f_{m}$ (MPa)	Composition of Infill Concrete Mixture Per 1 m^3^, kg						
			Cement 500	Medium-Sized Sand	Water	Granite Crushed Stone Fraction 5–10 mm	Expansion Additive	Softener	Infill Concrete Strength, $f_{c}$ (MPa)
P1	27.2	9.7	230	870	160	1040	–	1.80	14.0
P2			300	780	165	1040	–	2.34	19.5
P3			370	740	170	1000	–	2.88	26.5
P4			420	1530	210	–	–	3.24	17.0
P5			360	800	175	1000	60	2.52	19.0
P6			450	1610	215	–	60	3.06	22.0
P7			–	–	–	–	–	–	–

**Table 2:** Maximum loads and stresses of the prisms.

Specimen	$N_{crack}$ (kN)	$N_{max,mid}$ (kN)	$N_{max,mid}/N_{crack}$	$\sigma_{max}$ (MPa)
P1	378	840	0.45	24
P2	525	875	0.60	25
P3	693	925	0.75	26
P4	595	850	0.70	24
P5	656	875	0.75	25
P6	630	900	0.70	25
P7	562	625	0.90	27

**Table 4:** Comparison of experimental and theoretical values of the load capacity of masonry prisms.

Specimen	N_exp_ (kN) (Experimental Data)	N2 (kN) (Method 2)	N1 (kN) (Method 1)	N_exp_/N2	N_exp_/N1
P1	840	794	496	1.06	1.69
P2	875	860	690	1.02	1.26
P3	925	946	938	0.98	0.98
P4	850	830	602	1.02	1.41
P5	875	856	673	1.02	1.30
P6	900	891	779	1.01	1.16
P7	625	620	620	1.01	1.01

**Table 5:** Comparison of results.

Measured Value	HCB Masonry Prism with Concrete Infill			HCB Masonry Prism Without Concrete Infill		
	Test, P7	ANSYS	Difference %	Test, P1	ANSYS	Difference %
Longitudinal displacements of the specimens (mm)	1.58	1.480	6.3	1.560	1.620	3.8
Transverse displacements of the specimens (mm)	0.03	0.028	6.7	0.038	0.035	7.9
Stress, *σ* (MPa)	27.0	25.5	5.6	24.0	22.2	7.5

## Data Availability

The original contributions presented in the study are included in the article, further inquiries can be directed to the corresponding authors.
